# Metatranscriptomic Sequencing of Medically Important Mosquitoes Reveals Extensive Diversity of RNA Viruses and Other Microbial Communities in Western Australia

**DOI:** 10.3390/pathogens13020107

**Published:** 2024-01-25

**Authors:** Binit Lamichhane, Craig Brockway, Kimberly Evasco, Jay Nicholson, Peter J. Neville, Avram Levy, David Smith, Allison Imrie

**Affiliations:** 1 School of Biomedical Sciences, The University of Western Australia, Nedlands, WA 6009, Australia; binit.lamichhane@uwa.edu.au; 2 Biological and Applied Environmental Health Hazards, Department of Health, Perth, WA 6000, Australia; craig.brockway@health.wa.gov.au (C.B.); kimberly.evasco@health.wa.gov.au (K.E.); jay.nicholson@health.wa.gov.au (J.N.); peter.neville@health.wa.gov.au (P.J.N.); 3 PathWest Laboratory Medicine, Nedlands, WA 6009, Australia; avram.levy@health.wa.gov.au (A.L.); david.smith@health.wa.gov.au (D.S.); 4 UWA Medical School, The University of Western Australia, Nedlands, WA 6009, Australia

**Keywords:** metatranscriptomics, mosquitoes, virome, Western Australia, phylogeny

## Abstract

Mosquitoes harbor a wide diversity of microorganisms, including viruses that are human pathogens, or that are insect specific. We used metatranscriptomics, an unbiased high-throughput molecular approach, to describe the composition of viral and other microbial communities in six medically important mosquito species from across Western Australia: *Aedes vigilax*, *Culex annulirostris*, *Cx. australicus*, *Cx. globocoxitus*, *Cx. pipiens* biotype *molestus*, and *Cx. quinquefasciatus*. We identified 42 viral species, including 13 novel viruses, from 19 families. *Culex* mosquitoes exhibited a significantly higher diversity of viruses than *Aedes* mosquitoes, and no virus was shared between the two genera. Comparison of mosquito populations revealed a heterogenous distribution of viruses between geographical regions and between closely related species, suggesting that geography and host species may play a role in shaping virome composition. We also detected bacterial and parasitic microorganisms, among which *Wolbachia* bacteria were detected in three members of the *Cx. pipiens* complex, *Cx. australicus*, *Cx. pipiens* biotype *molestus*, and *Cx. quinquefasciatus*. In summary, our unbiased metatranscriptomics approach provides important insights into viral and other microbial diversity in Western Australian mosquitoes that vector medically important viruses.

## 1. Introduction

Mosquitoes (Diptera: Culicidae) are vectors of disease-causing agents that infect humans and animals. The major mosquito-borne diseases that are associated with significant public health burdens include malaria, dengue, Zika, West Nile, Japanese encephalitis, and chikungunya [1]. In addition, mosquitoes harbor an extensive diversity of viruses and other microorganisms. Although these microorganisms are often insect specific and have no direct impact on public health, they may act as natural competitors to the pathogens and affect their transmission [2]. Increasing evidence that insect-specific viruses may enhance or suppress the replication of medically important arboviruses co-infecting the same mosquito has highlighted the need to better understand insect-specific microorganisms and their potential applications [3,4,5,6].

Traditionally, microbial discovery relied on tissue culture, electron microscopy, and serological methods. These techniques may reveal limited information regarding microbial diversity and are often laborious and time-consuming. In recent years, with advances in next-generation sequencing techniques, metatranscriptomics has evolved as a cost-effective and reliable approach to reveal microbial diversity within mosquito populations [7,8], enhancing disease surveillance by rapidly detecting known pathogens [9]. Metatranscriptomics has enhanced the discovery of novel microbial agents and assessment of their evolutionary history, origin, and potential to cause disease [7,10,11].

In Western Australia (WA), mosquitoes are routinely tested for medically important arboviruses as part of longstanding, ongoing arbovirus surveillance programs undertaken by the WA Department of Health throughout the state [12,13]. Mosquitoes are routinely captured in the field and processed for arbovirus identification by RT-PCR, with virus isolation and sequencing of a selection of PCR positive samples [14]. In addition, Murray Valley encephalitis virus (MVEV), Japanese encephalitis virus (JEV), and West Nile Virus Kunjin subtype (KUNV) activity in northern WA is monitored via sentinel chicken serosurveillance [15]. Successful virus isolation depends on the capacity of a virus to grow in the given cell line, limiting detection to viruses with tropism for those cells. In addition, virus cross-reactivity in serological assays is a well-recognized issue in species identification [16]. Although the scope of detection has been broadened by using more generic PCR primers, for example pan-flavivirus primers to detect a range of flaviviruses, insect-specific and novel viruses are not identified by this approach. In addition, PCR inhibitors may produce false negative results [17]. The metatranscriptomic approach overcomes the drawbacks of traditional methods and allows the detection of a wide range of viruses, increasing confidence in arbovirus surveillance and virus discovery.

The application of metatranscriptomics has led to the identification of several novel viruses in Australia by directly sequencing pooled mosquito homogenates [11,14,18], and by sequencing mosquito homogenate cell culture supernatant [19,20]. The virus species identified were from a diverse range of viral families including *Bunyaviridae*, *Reoviridae*, *Rhabdoviridae*, *Flaviviridae*, *Iflaviridae*, *Orthomyxoviridae*, *Mesoniviridae*, *Mononegavirales*, *Nodaviridae*, *Phasmaviridae*, *Phenuiviridae*, *Picornavirales*, *Togaviridae*, *Tombusviridae*, *Totiviridae*, *Qinviridae*, *Ophioviridae*, *Luteoviridae*, *Narnaviridae*, *Partitiviridae*, and *Chrysoviridae* [11,14,18,19,20]. Mosquitoes clearly carry viruses from many genetically diverse virus families and groups.

In the present study we used a total-infectome approach to describe RNA viruses and other microbial communities in medically important mosquitoes using high-resolution meta-transcriptomics. We selected six medically important mosquito species for assessment of the WA mosquito infectome, including *Aedes vigilax*, *Culex annulirostris*, and four members of the *Culex pipiens* complex, namely *Cx. pipiens* biotype *molestus, Cx. quinquefasciatus,* and two Australian endemic complex members, *Cx. australicus* and *Cx. globocoxitus*.

*Ae. vigilax* and *Cx. annulirostris* are well-described vectors of Ross River virus (RRV) and Barmah Forest virus (BFV) [21,22]. Among members of *Cx. pipiens* complex in Australia, viruses including RRV, BFV, and MVEV have been detected in field-collected specimens [23]. *Cx. Quinquefasciatus* and *Cx. Pipiens* biotype *molestus* are known to vector WNV, St Louis encephalitis virus (SLEV), Usutu virus, avian malaria, and filariasis outside Australia [24,25].

We performed phylogenetic analyses and determined the abundance, type, number, and host association of each viral species in relation to other microorganisms present in the mosquito samples. This is the first study reporting the infectome of all Australian members of *Cx. pipiens* complex, and where geographical comparison of *Ae. vigilax* and *Cx. annulirostris* viromes was undertaken. We describe a diverse microbiome within these common WA mosquitoes, including 13 novel viral species.

## 2. Materials and Methods

### 2.1. Mosquito Samples

The mosquitoes analyzed in this study were collected by the Medical Entomology team of the Department of Health, Western Australia between 2018–2021 as part of the routine arbovirus surveillance program. Mosquitoes were collected using EVS CO_2_ traps [26] baited with dry ice and set at each location for approximately 12 h. The mosquitoes were euthanized upon trap collection by placing them on dry ice, transferred to labelled vials and transported to the Department of Health Medical Entomology laboratory in Perth, on dry ice to maintain a cold chain. Upon arrival, samples were stored at −80 °C until further processing.

**Figure 1:**
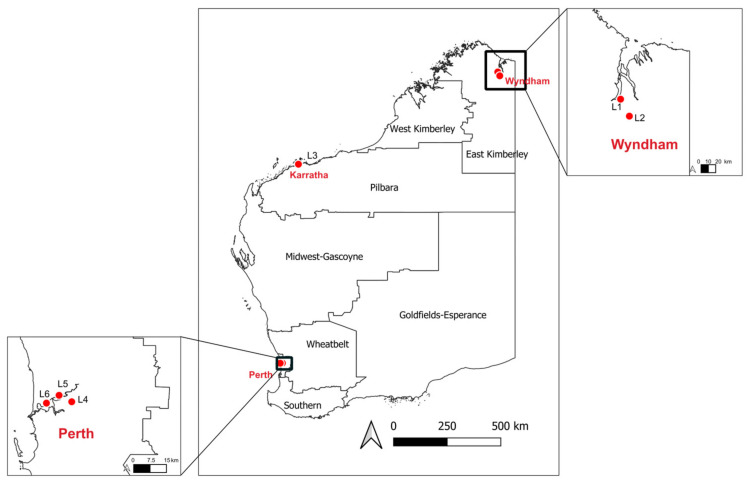
Geographic location of mosquito samples analyzed in this study. Locations of six sampling sites in Western Australia are marked with solid red dots.

Initial identification of mosquito species was completed using established morphological identification keys [27]. Eight pools of between 20–50 mosquitoes, each consisting of a single species collected at a single location on the same day, were prepared for meta-transcriptomic sequencing and analysis. The samples were from six trapping locations (L1–L6) across Wyndham, Karratha, and Perth (Figure 1). The species and the number of mosquitoes included in each pool are presented in Table 1. Morphological identification was verified by pulling legs from one mosquito from each pool and sequencing the Cytochrome oxidase I (COI) gene. As COI is not reliable in differentiating the species of *Cx. pipiens* complex members, detailed morphological diagnostic features based on work by Liehne on Western Australian mosquitoes were used for the speciation [27].

### 2.2. Sample Processing and Sequencing

A total of eight pools of mosquitoes were processed. The mosquito pools were placed in a 5 mL tube along with five glass beads (5 mm each) and 600 µL lysis buffer and homogenized in a Spex Mixer Mill (Spex SamplePrep, Metuchen, NJ, USA). Total RNA was extracted using an RNeasy Plus universal mini kit (QIAGEN, Hilden, Germany) following the manufacturer’s instructions. The quality and quantity of RNA were evaluated using LabChip GX (PerkinElmer, Waltham, MA, USA). An RNA sequencing library was constructed using a TruSeq RNA Library Prep kit (Illumina, San Diego, CA, USA) with ribosomal RNA depletion using a Ribo-Zero Plus rRNA depletion kit (Illumina). Paired-end (150 bp) sequencing of the dual-indexed libraries was performed on a NovaSeq platform (Illumina). All library preparation and sequencing were performed by the Australian Genome Research Facility (AGRF), Melbourne, Australia.

### 2.3. Virus Discovery

Demultiplexed sequencing reads were trimmed for quality and adapter removal using the BBTools software package (https://jgi.doe.gov/data-and-tools/software-tools/bbtools/; accessed on 1 July 2022). The resulting clean reads were assembled de novo using Megahit v 1.2.9 [28]. Contigs were then compared against the NCBI Genbank non-redundant protein (nr) database using Diamond blastx v 2.0.13.151 [29] with an expected −value threshold of 1 × 10^−5^ to remove false positives. Host contigs misidentified as viruses were removed by comparing them with the Genbank non-redundant nucleotide database (nt). The reads were subsequently mapped back to contigs using Bowtie2 v2.3 [30] and corrected for mis-assembly by visualizing the results in IGV [31]. Novel viral species were identified as those with <90% RdRp amino acid identity to previously described viruses [9].

Viral abundance was determined using the formula “total viral reads/total non-rRNA reads * 1 million” as RPM (reads per million) [11,32]. In addition, contigs for a virus with frequency <0.1% of the most abundant reads of that virus in a different library were removed as potential contamination due to index-hopping.

### 2.4. Viral Genome Characterization and Phylogenetic Analysis

After identifying potential viral contigs, the ORF and deduced amino acid sequence were determined by using Geneious Prime 2022.0.1 and compared with the closest reference genomes from the NCBI viral genome database. The function of the ORFs was determined by homology with known viral proteins from the closest reference genome.

We utilized RNA-dependent RNA polymerase (RdRp) sequences for the phylogenetic analysis. We included RdRp sequences from previously reported viruses which have some identity to the queried virus. We also included RdRp of previously described mosquito viruses in the same family for our analysis. Protein sequences were aligned using the E-INS-I algorithm in MAFFT v7.450 [33]. Poorly aligned regions were removed by using trimAl v1.4 [34]. The best fit model of the amino acid substitution was calculated using ProtTest v3.4 [35]. Phylogenetic trees were constructed using RaxML with 1000 bootstrap replicates and visualized by FigTree v1.4.4 (https://tree.bio.ed.ac.uk/software/figtree/, accessed on 12 December 2022).

### 2.5. Identification of Non-Viral Microbes

To identify non-viral microbial communities, Metaphlan4 [36] was employed. The marker gene *groEL* was used for the phylogenetic inference of *Wolbachia* [37]. We estimated the abundance of the microorganisms in positive samples by mapping the reads to the corresponding reference whole genomes to estimate RPM. To rule out possible bias in abundance estimation, highly conserved rRNA genes were removed from the reference genomes before the mapping.

### 2.6. Accession Numbers

Viral genomic sequences generated from this study were deposited in the NCBI Genbank under the accession numbers PP066138 to PP066243. The raw sequencing datasets are available in the NCBI Sequence Read Archive repository under the BioProject ID PRJNA1059154.

## 3. Results

### 3.1. Mosquito Virome

**Figure 2:**
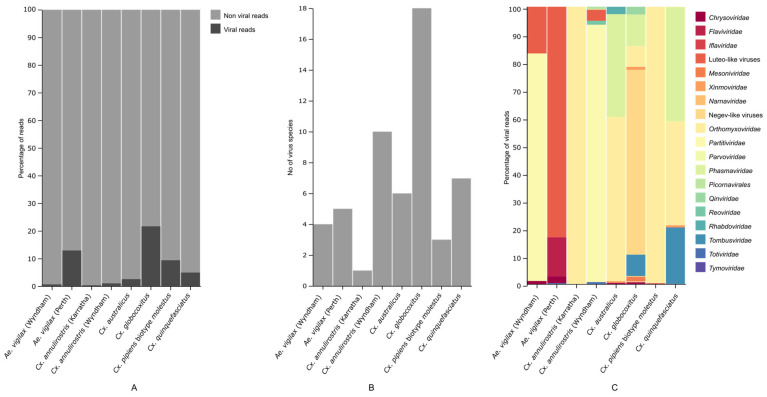
Diversity and abundance of total virome within each mosquito species, by location. The figure shows in each mosquito pool, (**A**) the proportion of viral and non-viral reads, (**B**) the number of virus species found, and (**C**) the proportion of viral RNA reads in each viral group.

We characterized the RNA viral transcriptome of eight pools of mosquitoes belonging to six mosquito species that are common in Western Australia. High throughput sequencing resulted in 84.5 million to 183.8 million reads per pool, which led to 41,679 to 135,094 contigs after de novo assembly (Table S1). The viral reads compared to the total non-viral rRNA depleted reads per library varied between 0.35 to 21.7 (Figure 2A). On analyzing the contigs, we identified 42 virus species. There were 10 negative-sense single-stranded RNA (ssRNA); 19 positive-sense ssRNA; 12 double-stranded RNA; and one ssDNA viral sequence. The number of virus species in each library ranged from one to 18 (Figure 2B). The viruses fell into a diverse range of RNA virus orders and families, namely *Xinmoviridae*, *Orthomyxoviridae*, *Rhabdoviridae*, *Mesoniviridae*, *Flaviviridae*, *Iflaviviridae*, *Picornavirales*, *Phasmaviridae*, *Tombusviridae*, *Reoviridae*, *Narnaviridae*, *Totiviridae*, *Qinviridae*, *Tymoviridae*, *Partitiviridae*, *Chrysoviridae*, and *Densoviridae* as well as representatives from the highly divergent group of viruses namely Negev-like viruses and Luteo-like viruses (Figure 2C). None of the viruses were found in the host genome, excluding the likelihood of these viruses being present as endogenous viral elements (EVEs).

The abundance of each virus as measured by RPM varied from 5.60 to 85,573.19 (Table 2). In comparison to the viruses, the abundance of host COI gene reads varied from 5356.84 to 32,205.12 RPM.

### 3.2. Characterization of Virus Diversity

#### 3.2.1. Double-Stranded RNA Viruses

**Figure 3:**
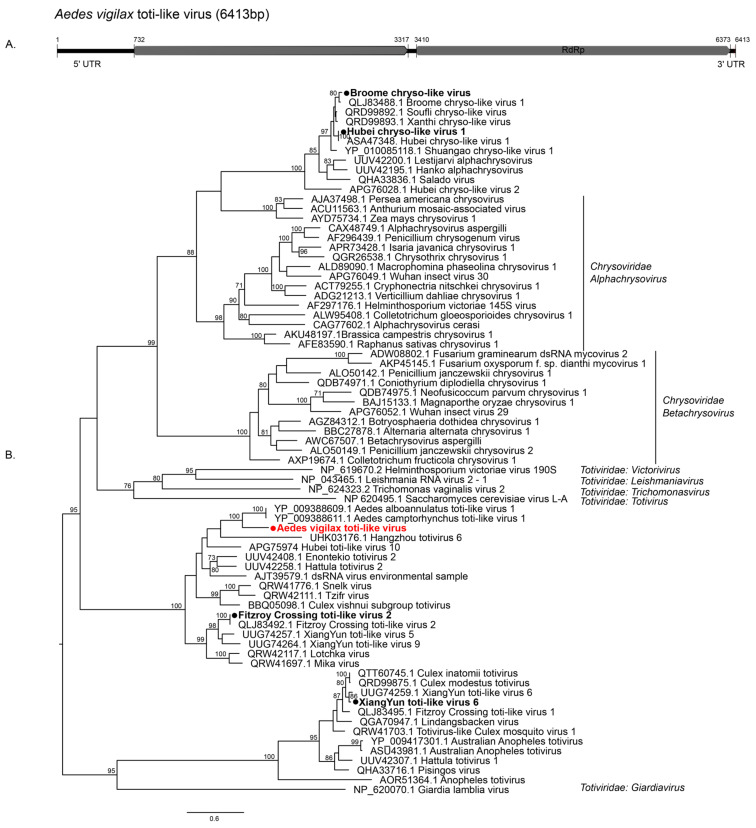
Diversity and genomic features of viruses identified within the family *Totiviridae* and *Chrysoviridae.* (**A**) Genomic features of novel AVTLV detected in this study. (**B**) Midpoint rooted maximum likelihood phylogenetic tree of toti-chryso cluster showing the position of novel viruses (represented by solid red circles) and new variant identified (represented by solid black circles). Bootstrap support values are displayed when greater than 70.

We identified 12 double-stranded RNA viruses which fell within *Partitiviridae* (*n* = 6) *Totiviridae* (*n* = 3), *Chrysoviridae* (*n* = 2), and *Reoviridae* (*n* = 1). We detected one novel totivirus, tentatively named *Aedes vigilax* toti-like virus (AVTLV), consisting of an unsegmented genome with two ORFs (Figure 3A). AVTLV clustered with *Aedes camptorynchus* toti-like virus with 56% RdRp amino acid identity, which was also identified from a mosquito (Figure 3B) [11]. XiangYun toti-like virus 6 (XYTLV6) and Fitzroy Crossing toti-like virus (FCTLV2) previously detected from mosquitoes in China and WA [14], respectively, were also identified in this study. Both the chrysoviruses identified in the present study, Broome chryso-like virus (BCLV1) and Hubei chryso-like virus 1 (HCLV1), were previously found in mosquitoes [11,14].

**Figure 4:**
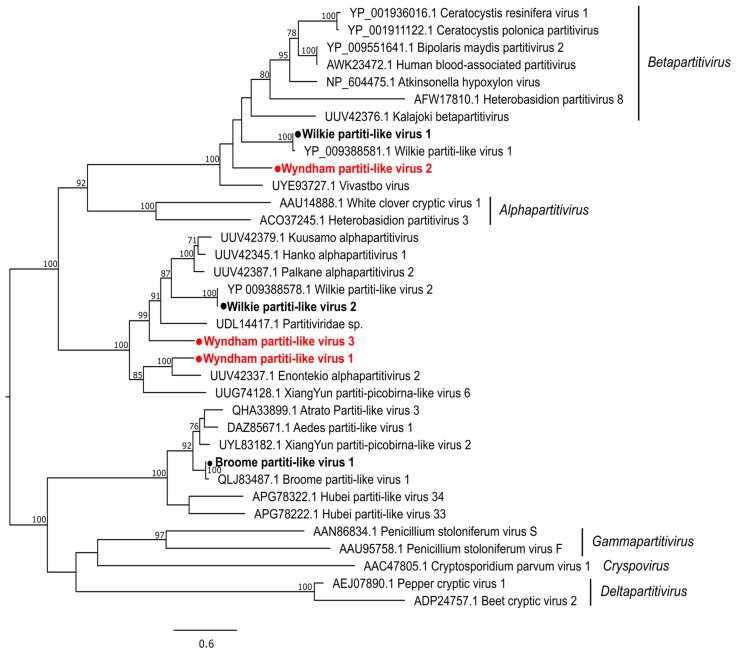
Diversity of viruses identified within the family *Partitiviridae*. Midpoint rooted maximum likelihood phylogenetic tree showing the position of novel viruses (represented by solid red circles) and new variants identified (represented by solid black circles). Bootstrap support values are displayed when greater than 70.

Of the three novel partiti-like viruses, Wyndham partiti-like virus 1 (WYPLV1) clustered with the viruses sequenced from mosquitoes in Finland and China. WYPLV2 and WYPLV3 did not cluster with any viruses sequenced from the mosquito host but were grouped with viruses discovered from other arthropod hosts and environmental samples (Figure 4). We also detected Wilkie partiti-like virus 1 (WPLV1), WPLV2 and Broome partiti-like virus 1 (BPLV1) previously detected in WA mosquitoes. While WPLV2 and BPLV1 cluster with viruses identified from mosquitoes, WPLV1 groups mainly with the viruses identified from fungi (Figure 4). The only reovirus identified, Broome reo-like virus (BRLV), previously identified from *Cx. annulirostris* in WA [14], was also detected from *Cx. annulirostris* in the present study, thus validating previous findings. BRLV forms a sister clade to the genus *Fijivirus* of the subfamily *Spinareovirinae* (Figure S1) and clusters with the virus identified from water striders (family *Gerridae*) and in river sediment in China [10,38].

#### 3.2.2. Positive-Sense Single-Stranded RNA Viruses

In total, we identified 19 positive-sense ssRNA viruses, belonging to nine virus groups: including Luteo-sobemo cluster (*n* = 5), *Narnaviridae* (*n* = 4), Negev-like (*n* = 4), *Orthoflavivirus* (*n* = 1), *Iflaviridae* (*n* = 1), *Mesoniviridae* (*n* = 1), *Picornavirales* (*n* = 1), *Tombusviridae* (*n* = 1) and *Tymoviridae* (*n* = 1); including seven that are novel.

**Figure 5:**
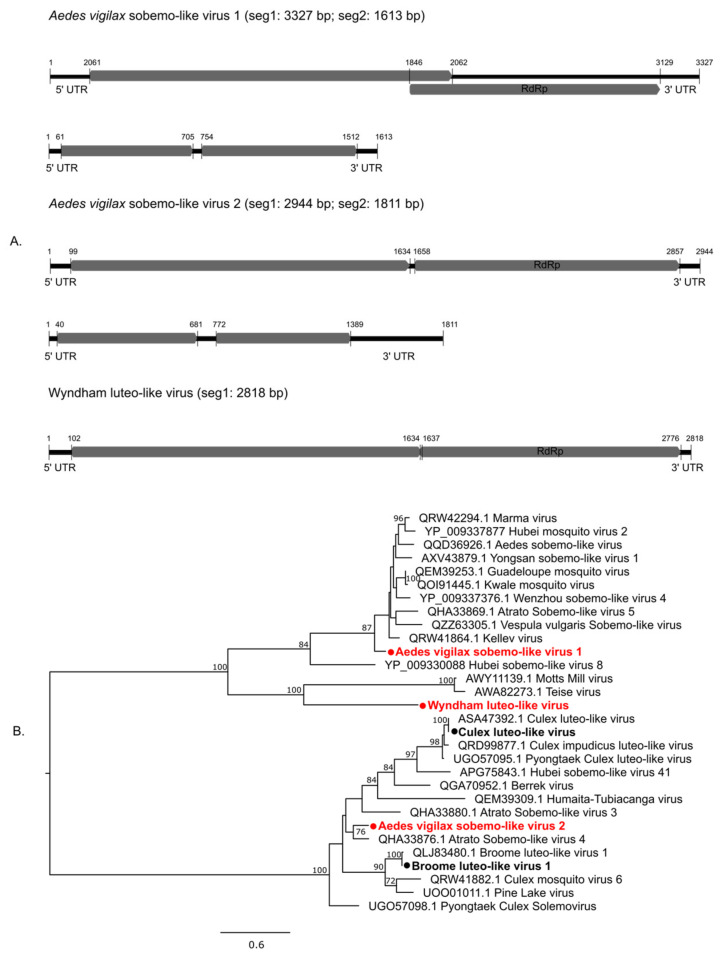
Diversity of viruses identified within the Luteo-sobemo-like viruses (**A**) Genomic features of novel AVSLV1 and AVSLV2 detected in this study. (**B**) Midpoint rooted maximum likelihood phylogenetic tree of luteo-sobemo group showing the position of novel viruses (represented by solid red circles) and new variants identified (represented by solid black circles). Bootstrap support values are displayed when greater than 70.

The Luteo-sobemo group comprises members associated with a diverse host group. They are primarily plant viruses but have recently been found in molluscs, protists, nematodes, and arthropods [10], including mosquitoes [11]. Three novel members of this group were identified in this study: *Aedes vigilax* sobemo-like virus 1 (AVSLV1), AVSLV2, and Wyndham luteo-like virus (WYLLV). The bi-segmented genomes of AVSLV1 and AVSLV2 are presented in Figure 5A, showing corresponding ORFs. AVSLV1 and AVSLV2 clustered with mosquito-associated viruses, while WYLLV clustered with viruses detected in *Drosophila* (Figure 5B).

**Figure 6:**
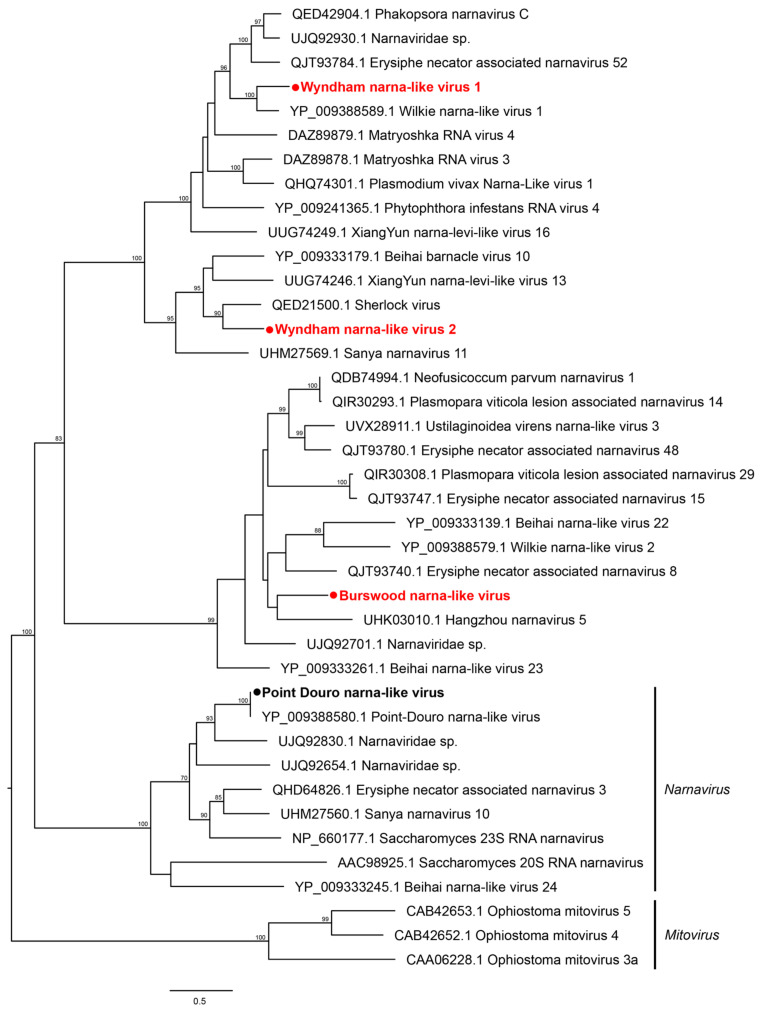
Diversity of viruses identified within the family *Narnaviridae* Midpoint rooted maximum likelihood phylogenetic tree showing the position of novel viruses (represented by solid red circles) and new variant identified (represented by solid black circles). Bootstrap support values are displayed when greater than 70.

**Figure 7:**
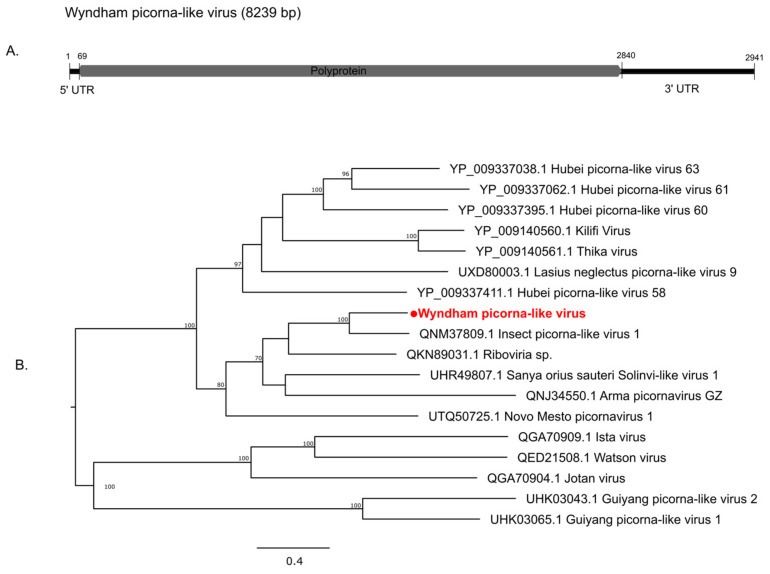
Diversity of viruses identified within the order *Picornavirales* (**A**) Genomic features of novel virus WYPLV (**B**) Midpoint rooted maximum likelihood phylogenetic tree showing the position of WYPLV (represented by solid red circle). Bootstrap support values are displayed when greater than 70.

The genome organization containing a single RdRp gene of the narnaviruses is shown in Figure 6. None of the four narnaviruses detected in this study clustered with mosquito-associated viruses. Wyndham narna-like virus 1 (WYNLV1) clustered with fungal viruses while WYNLV2 was grouped with a virus sequenced from an Australian flea. Burswood narna-like virus (BNLV) clustered with Hangzhou narnavirus 5 sequenced from flea beetles collected from rice fields in China (Figure 6). The abundance levels of WYNLV1 and WYNLV2 were very low (i.e., <10 RPM), suggesting their very weak association with the mosquito host (Table 2). The genome segment of the only member of *Picornavirales* detected in this study, Wyndham picorna-like virus (WYPILV), showing the polyprotein is shown in Figure 7A. WYPILV clustered with Insect picorna-like virus 1 initially detected in *Thrips tabaci* from Italy (Figure 7B).

We detected a strain of Parramatta River virus (PARV), an insect-specific flavivirus, in *Ae. vigilax* trapped in Perth. This WA PARV variant shared 96% RdRp amino acid identity with PARV isolated from *Ae. vigilax* in 2007 in Sydney [39] (Figure S2). We also identified previously described viruses, which were found to be associated with mosquitoes: Alphamesonivirus 1 Ngewotan strain (AMNV) (Figure S3), Culex Iflavi-like virus 1 (CILV1) (Figure S4), Culex-associated Tombus-like virus (CATLV) (Figure S5), Guadeloupe Culex tymo-like virus (GCTLV) (Figure S6), Culex negev-like virus 1 (CNLV1), CNLV2, CNLV3, and Cordoba virus (CV) (Figure S7).

#### 3.2.3. Negative-Sense Single-Stranded RNA Viruses

**Figure 8:**
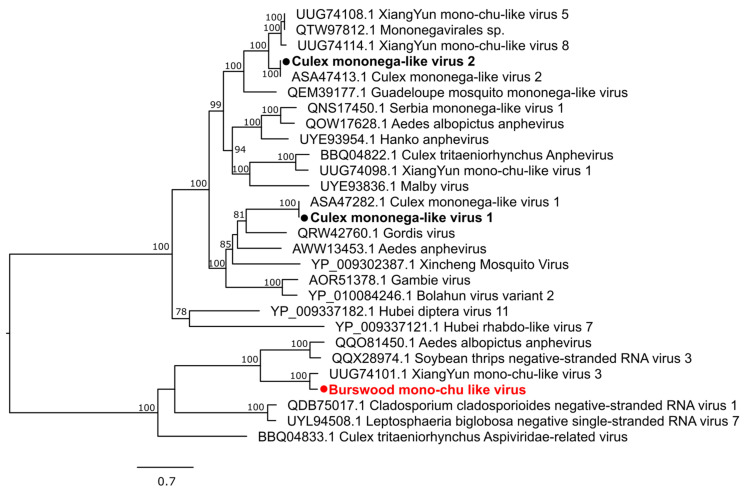
Diversity of viruses identified within the family *Xinmoviridae*. Midpoint rooted maximum likelihood phylogenetic tree showing the position of novel virus (represented by solid red circle) and new variants identified in the present study (represented by solid black circles). Bootstrap support values are displayed when greater than 70.

We identified 10 negative-sense ssRNA viruses, including one novel virus, that belong to five virus groups: *Xinmoviridae* (*n* = 3) *Orthomyxoviridae* (*n* = 3), *Phasmaviridae* (*n* = 1), *Qinviridae* (*n* = 2), and *Rhabdoviridae* (*n* = 1). The family *Xinmoviridae* has recently been included in the order *Mononegavirales* and contains viruses reported from insects [40]. We identified two known viruses and a novel virus that are members of this family. Culex mononega-like virus 1 (CMLV1) and CMLV2, both previously reported from mosquitoes, were also identified. The novel Burswood mono-chu-like virus clustered with the XiangYun mono-chu-like virus 3 detected in mosquitoes from China (Figure 8).

Three members of the family *Orthomyxoviridae*, namely Wuhan mosquito virus 3 (WMV3), WMV4, and WMV6 were identified (Figure S8). WMV6 was previously detected in WA mosquitoes and was shown to have a strong association with the mosquito host. We found WMV6 in all *Cx. pipiens* complex pools. WMV3 showed 91% RdRp amino acid similarity to WMV3 detected from *Cx. tritaeniorhynchus* in China [41] and was on the borderline of being novel based on our threshold of <90% RdRp amino acid identity.

The Culex phasma-like virus (CPLV) and Culex rhabdo-like virus (CRLV) detected in our study clusters clearly with mosquito-associated viruses (Figures S9 and S10) supporting previous reports [11]. *Qinviridae* is a recently described family [42] with member viruses that are associated with arthropods [10]. We report two members of this family, Wilkie qin-like virus 1 (WQLV1) and Fitzroy Crossing qinvirus 1 (FCQV1), both clustering with viruses previously sequenced from mosquitoes (Figure S11). Although the association of WQLV1 with mosquito hosts has been questioned [11], two recent studies have sequenced related viruses from mosquitoes sampled elsewhere [32,43], thus strengthening support for association with mosquito hosts.

#### 3.2.4. Single-Stranded DNA Virus

**Figure 9:**
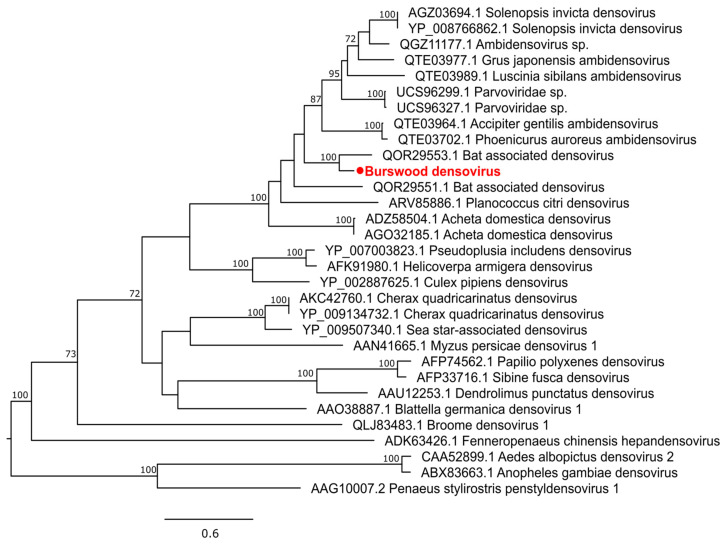
Midpoint rooted maximum likelihood phylogenetic tree of Burswood densovirus (represented by solid red circle). Bootstrap support values are displayed when greater than 70.

A novel densovirus (Family: *Parvoviridae*) was detected from *Cx. quinquefasciatus* which we tentatively name Burswood densovirus. It shares 71% NS1 amino acid identity and clusters with a Bat associated densovirus sequenced from bat droppings in the USA (Figure 9). Two ORFs were present, one fully encoding NS1 protein and the other partially encoding VP1.

### 3.3. Geographical Differences in Aedes vigilax and Culex annulirostris Virome

**Figure 10:**
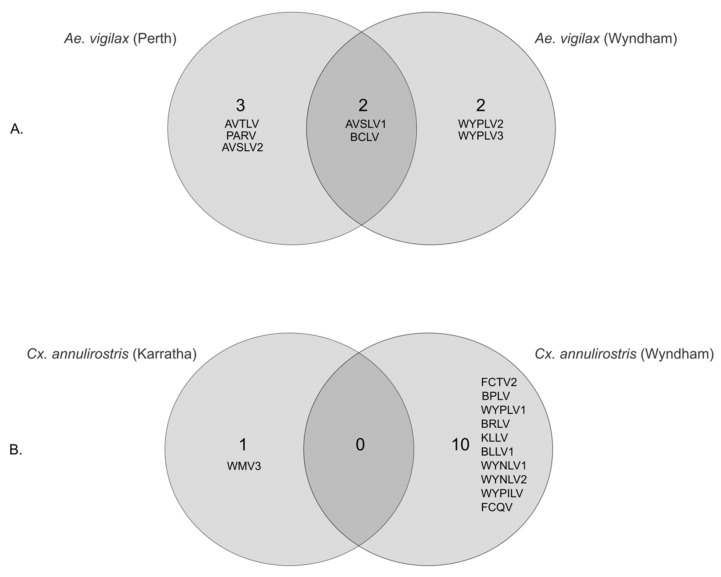
Venn diagrams showing shared and unique viruses across different geographical locations in (**A**) *Ae. vigilax* and (**B**) *Cx. annulirostris*.

Of the seven viral species identified in *Ae. vigilax*, mosquito pools sampled in Wyndham in the far north of WA, and in Perth in the south of WA, harbored four and five viral species, respectively, including two viruses (BCLV1 and AVSLV1) shared in both pools (Figure 10). BCLV1 in the Perth pool was in high abundance (RPM 3374) compared to the Wyndham pool (RPM 331) (Table 2). PARV, AVSLV1, and AVSLV2 were present at very high abundance (>10,000 RPM) in the Perth samples, suggesting their strong association with this mosquito host. Apart from BCLV1 and PARV, all five viruses described in *Ae. vigilax* were novel. The RPM values of all four viruses from Wyndham were found to be considerably low as compared to the host COI (Table 2).

There was a significant difference in the *Cx. annulirostris* virome between Karratha and Wyndham, locations in far northern WA, approximately 1300 km apart. The mosquito pool sampled in Wyndham harbored 10 viruses, while the pool from Karratha harbored only one virus; no viruses were shared between the two sampling sites (Figure 9). The one virus species in the Karratha pool, WMV3, belongs to the family *Orthomyxoviridae*. Three viral species, two *Narnaviridae* (WYNLV1 and WYNLV2) and one *Picornaviridae* (WYPILV), found in *Cx. annulirostris* were novel. Only BPLV1 detected from the Wyndham pool had RPM values higher than the host COI (Table 2).

### 3.4. Non-Viral Microorganisms

Analysis of the non-viral reads in our samples identified two protists: *Cystoisospora* and *Trypanosoma*, and three bacteria: *Entomospira culicis*, *Zymobacter palmae*, and *Wolbachia* (Table 2). *Cystoisospora* was detected in *Ae. vigilax* from Wyndham and *Cx. annulirostris* from both Karratha and Wyndham, while being absent in all five pools from Perth. *Trypanosoma* was detected in seven pools except *Ae. Vigilax* from Perth (Table 2). No fungi were detected in our analysis.

**Figure 11:**
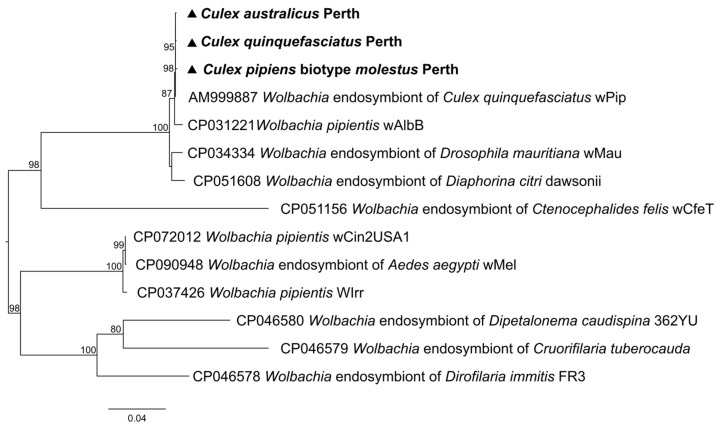
Phylogenetic tree based on the *groEL* gene showing the position of *Wolbachia* from *Cx. australicus*, *Cx. quinquefasciatus*, and *Cx. pipiens* biotype *molestus* as indicated by ▲.

*Wolbachia* had relatively high abundance levels in three of our *Cx. pipiens* complex members, *Cx. Australicus* (RPM 997), *Cx. Quinquefasciatus* (RPM 3342), and *Cx. pipiens* biotype *molestus* (RPM 2953). The *Wolbachia* identified clustered closely with the *Wolbachia* endosymbiont of *Cx. quinquefasciatus* Pel strain wPip (Genbank Accession no: AM999887) with *groEL* gene identity of 99.2% (Figure 11). Our results suggest that the presence or absence, and the abundance levels, of *Wolbachia* have not affected viral abundance and diversity.

## 4. Discussion

Arbovirus surveillance and discovery have initially depended on the use of suckling mice [44] and more recently on cell culture techniques to isolate viruses from insect homogenates, along with detection of virus-specific antibodies [45]. Due to the selective nature of these methods, only a limited number of viruses were identified. However, recent advancements in metatranscriptomic sequencing have significantly accelerated the discovery of new viruses and have the potential to greatly enhance arbovirus surveillance programs.

We used a high throughput metatranscriptomic approach to investigate viruses, bacteria, and eukaryotic microorganisms in six species of mosquitoes commonly found in WA. None of the 42 virus species we identified was a human pathogen; most are likely to be ISVs, some of which may have a commensal role in their insect hosts. This finding is not surprising as many previous studies on mosquitoes known to vector medically important viruses have not identified human pathogens despite detecting several novel viruses [8,46,47,48]. Two metatranscriptomic investigations previously conducted in WA by our group also found that the identified viruses were associated with the mosquito host or with other microorganisms within the mosquito [11,14]. Other studies have detected vertebrate pathogens including Sindbis virus [32,43] and Japanese encephalitis virus [49] in field-collected mosquitoes, but the overall abundance was relatively low. It is possible that pathogenic viruses replicate at low levels in mosquitoes especially during inter-epidemic periods and thereby exhibit low prevalence and abundance. Human and animal viral pathogens in mosquito vectors likely comprise a very small fraction of the mosquito virome.

In the present study the virome of *Culex* mosquitoes exhibited greater viral diversity at the genus level than *Aedes* mosquitoes. We did not identify an overlap in virome composition between *Culex* and *Aedes,* as reported previously by other groups [50,51]. However, in an earlier study of mosquitoes trapped in WA in 2015, two viruses were shared among the *Culex* and *Aedes* populations [11]. ISVs demonstrate mosquito host preferences, even among ISV that belong to the same genus, [11,52] a trait that is observed among pathogenic arboviruses vectored by mosquitoes that feed on a range of vertebrate hosts [53]. Among *Culex* species, there was no overlap in virome between *Cx. annulirostris* and members of the *Cx. pipiens* complex. Although there was considerable virus sharing among members of the *Cx. pipiens* species complex as might be expected because of their close evolutionary relatedness [54], viral abundance and diversity were uneven across species. These differences may be attributed to the limited sample size in this study, differences in the local habitat of mosquitoes, and the variety of vertebrate hosts [32]. At the geographical level, the variation in abundance and diversity of mosquito populations is striking in *Cx. annulirostris* and *Ae. vigilax*. Williams et al. found uneven virome distribution and abundance among *Cx. annulirostris* populations sampled at sites that were relatively close together [14]. These findings collectively suggest that host and environmental factors have a significant role in shaping the virome of mosquito populations; however, detailed long-term longitudinal sampling is required to validate this further.

Our data complement the findings of earlier studies conducted in WA. Fifteen species belonging to 11 viral families identified in *Cx. pipiens* complex members collected in 2018, identified in the present study, were described in the same mosquito species in an analysis of 519 mosquitoes sampled in 2015 in the south-west region of WA [11]. We identified an additional five viral species, WMV4, BMCLV, CV, CTLV, and CATLV, in the 2018 *Cx. pipiens* complex species sampled in southern WA. Temporal factors may play a role in defining virome composition. Time series analyses conducted in China reported differences in virome composition [55,56].

Similarly, six viral species identified in *Cx. annulirostris* in the present study, collected in 2018, were reported in an earlier study conducted in mosquitoes also collected in 2018 in northern WA, in the same region but in different locations [14], thus validating these earlier findings and strengthening our understanding of the WA mosquito virome. In addition, we identified five novel viral species from *Cx. annulirostris*: WYPLV1, WYLLV, WYNLV1, WYNLV2, and WYPILV. Comparison among studies conducted in the same geographical region, in different time periods, contribute to our understanding of viral diversity, host associations, ecology, and evolution. However, factors including differences in sequencing methods, bioinformatic analysis techniques, and morphological criteria used for mosquito species identification should also be considered when making direct comparisons [32].

The non-targeted transcriptomes generated in the present study not only contain viral sequences, but also host genes and genes of other microorganisms cohabiting the host. The presence of *Wolbachia* bacteria in three members of *Cx. Pipiens* complex (*Cx. Australicus*, *Cx. Quinquefasciatus*, and *Cx.pipiens* biotype *molestus*) is in line with previous studies where *Wolbachia* was detected in most *Cx. pipens* members [57,58,59,60]. The *groEL* gene was almost identical in all three mosquito populations, as may be expected for closely related species collected from the same geographical area during the same period. Transmission of *Wolbachia* between the mosquito populations could occur due to feeding on the same plants [61], or through mites that feed on mosquitoes [62]. Detection of other microbes including *Trypanosoma*, *Zymobacter palmae*, and *Entomospira culicis* supports earlier studies of mosquitoes trapped in WA and elsewhere [11,63,64,65], and further work is needed to elucidate any possible roles in modulating mosquito-borne disease transmission.

In this study, we relied on morphological identification of *Cx. pipiens* complex mosquitoes as there is no PCR-based identification method available for discriminating Australian *Cx. pipiens* complex mosquitoes. *Cx. pipiens* complex mosquitoes are known to be morphologically similar, and genetic differentiation based on COI barcodes is unreliable. Although some studies [66,67] have used polymorphisms in the *ACE2* marker to discriminate among the members of *Cx. pipiens* complex, these haplotypes are largely regional, and no data exist for Australian *Cx. pipiens* complex. In the absence of genetic differentiation methods we followed standard morphological identification protocols that have been applied by experienced medical entomologists in WA since the 1970s.

A major limitation of our present study is the lack of replicate mosquito pools, to strengthen statistical confidence in microbe detection and to establish clear host associations. Only eight species of mosquitoes have so far been studied in WA, including in the present study. About 100 species of mosquitoes are found in Western Australia, and applying metatranscriptomics to characterize the virome of more species will increase our understating of viral evolution and diversity, and our understanding of cross-species transmission. Factoring in seasonal, geographic, and gender variation and metadata including temperature, humidity, and other environmental factors should be considered in future studies. While no evidence was found suggesting the virus sequences generated in this study are from endogenous viral elements, additional analysis and validation are needed to strengthen the robustness of the data. Finally, a major limitation of metatranscriptomics is that it does not distinguish active from inactivate members of the microbial community.

In summary, by using a high-throughput non-targeted metatranscriptomic approach we have demonstrated that we can reveal microorganisms, particularly viruses, in mosquito populations with great precision and depth.

## 5. Conclusions

We utilized a high-throughput non-targeted metatranscriptomic approach to illustrate the precision and depth with which microorganisms, notably viruses, can be identified within mosquito populations. We have developed a valuable repository of data on viral and other microbial communities in Western Australian mosquitoes that will support investigation of associations among mosquitoes, viruses, and their surrounding environments and foster a deeper understanding of mosquito infectomes.

## Figures and Tables

**Table 1:** Mosquito species analyzed in this study.

Mosquito Species	City	Location Code	GPS Coordinates	Number of Mosquitoes	Year of Collection
*Ae. vigilax*	Wyndham	L1	−15.4967, 128.1432	50	2018
*Ae. vigilax*	Perth	L6	−31.9863, 115.8272	50	2021
*Cx. annulirostris*	Wyndham	L2	−15.7143, 128.2558	50	2018
*Cx. annulirostris*	Karratha	L3	−20.7214, 116.83	50	2020
*Cx. australicus*	Perth	L4	−31.9797, 115.9501	20	2018
*Cx. globocoxitus*	Perth	L5	−31.9483, 115.8885	20	2018
*Cx. quinquefasciatus*	Perth	L5	−31.9483, 115.8885	20	2018
*Cx. pipiens* biotype *molestus*	Perth	L4	−31.9797, 115.9501	20	2018

**Table 2:** Individual abundance of each virus, host COI, and other microbes, by mosquito trapping location. The abundance is expressed as RPM.

Classification	Virus	*Aedes vigilax*		*Culex* *annulirostris*		*Culex* *quinquefasciatus*	*Culex pipiens* biotype *molestus*	*Culex* *australicus*	*Culex* *globocoxitus*
		Perth	Wyndham	Karratha	Wyndham	Perth	Perth	Perth	Perth
*Totiviridae*	AVTLV	445.21	0.00	0.00	0.00	0.00	0.00	0.00	0.00
	FCTLV2	0.00	0.00	0.00	74.40	0.00	0.00	0.00	0.00
	XYTLV6	0.00	0.00	0.00	0.00	36.67	0.00	0.00	0.00
*Chrysoviridae*	BCLV1	3374.37	331.94	0.00	0.00	0.00	0.00	0.00	0.00
	HCLV1	0.00	0.00	0.00	0.00	0.00	0.00	114.29	1166.24
*Partitiviridae*	BPLV1	0.00	0.00	0.00	9645.40	0.00	0.00	0.00	0.00
	WYPLV1	0.00	0.00	0.00	10.30	0.00	0.00	0.00	0.00
	WYPLV2	0.00	323.44	0.00	0.00	0.00	0.00	0.00	0.00
	WYPLV3	0.00	304.67	0.00	0.00	0.00	0.00	0.00	0.00
	WPLV1	0.00	0.00	0.00	0.00	0.00	0.00	0.00	128.81
	WPLV2	0.00	0.00	0.00	0.00	0.00	0.00	0.00	310.23
*Reoviridae*	BRLV	0.00	0.00	0.00	148.40	0.00	0.00	0.00	0.00
*Flaviviridae*	PARV	19,106.13	0.00	0.00	0.00	0.00	0.00	0.00	0.00
*Mesoniviridae*	AMNV	0.00	0.00	0.00	0.00	0.00	0.00	0.00	2301.93
*Iflaviridae*	CILV1	0.00	0.00	0.00	0.00	0.00	301.64	0.00	0.00
*Luteo-like*	AVSLV1	36,925.01	5035.38	0.00	0.00	0.00	0.00	0.00	0.00
	AVSLV2	75,784.76	0.00	0.00	0.00	0.00	0.00	0.00	0.00
	WYLLV	0.00	0.00	0.00	6.90	0.00	0.00	0.00	0.00
	CLLV	0.00	0.00	0.00	0.00	0.00	0.00	0.00	399.07
	BLLV1 ^a^	0.00	0.00	0.00	402.30	0.00	0.00	0.00	0.00
*Narnaviridae*	BNLV	0.00	0.00	0.00	0.00	0.00	0.00	0.00	224.80
	WYNLV1	0.00	0.00	0.00	5.60	0.00	0.00	0.00	0.00
	WYNLV2	0.00	0.00	0.00	9.00	0.00	0.00	0.00	0.00
	PDNLV ^a^	0.00	0.00	0.00	0.00	0.00	0.00	0.00	515.69
*Picornaviridae*	WYPILV	0.00	0.00	0.00	83.00	0.00	0.00	0.00	0.00
*Tombusviridae*	CATLV	0.00	0.00	0.00	0.00	8038.81	0.00	0.00	13,463.94
*Tymoviridae*	GCTLV	0.00	0.00	0.00	0.00	43.23	0.00	0.00	0.00
Negev-like	CNLV1	0.00	0.00	0.00	0.00	0.00	0.00	0.00	11,188.65
	CNLV2	0.00	0.00	0.00	0.00	0.00	0.00	0.00	81,237.81
	CNLV3	0.00	0.00	0.00	0.00	0.00	0.00	0.00	49,987.88
	CV	0.00	0.00	0.00	0.00	0.00	0.00	0.00	16,348.41
*Xinmoviridae*	BMCLV ^a^	0.00	0.00	0.00	0.00	0.00	0.00	0.00	236.76
	CMLV1	0.00	0.00	0.00	0.00	298.40	0.00	160.76	1623.34
	CMLV2	0.00	0.00	0.00	0.00	0.00	0.00	0.00	39.35
*Orthomyxoviridae*	WMV3	0.00	0.00	3523.33	0.00	0.00	0.00	0.00	0.00
	WMV4	0.00	0.00	0.00	0.00	0.00	8295.23	225.96	0.00
	WMV6	0.00	0.00	0.00	0.00	14,885.72	85,573.19	14,975.02	13,039.50
*Phasmaviridae*	CPLV	0.00	0.00	0.00	0.00	16,355.87	0.00	9495.19	19,896.30
*Qinviridae*	FCQV1	0.00	0.00	0.00	17.60	0.00	0.00	0.00	0.00
	WQLV	0.00	0.00	0.00	0.00	0.00	0.00	0.00	4843.81
*Rhabdoviridae*	CRLV	0.00	0.00	0.00	0.00	0.00	0.00	684.93	0.00
*Parvoviridae*	BDNV ^a^	0.00	0.00	0.00	0.00	808.23	0.00	0.00	0.00
Host COI		20,256.85	32,205.12	17,267.12	9052.50	10,366.23	12,927.79	11,573.13	5356.84
*Wolbachia*		0.00	0.00	0.00	0.00	2953.62	3341.82	997.00	0.00
*Entomospira culicis*		0.00	0.00	85.99	0.00	0.00	0.00	39.09	0.00
*Zymobacter palmae*		0.00	0.00	158.38	210.50	0.00	0.00	0.00	0.00
*Cystoisospora* sp.		0.00	36.89	17.90	11.31	0.00	0.00	0.00	0.00
*Trypanosoma* sp.		0.00	6.79	2.54	13.31	11.47	1.69	19.07	41.27

^a^ PDNLV: Point-Douro narna-like virus; BMCLV, Burswood mono-chu-like virus; BDNV: Burswood densovirus; BLLV1: Broome luteo-like virus 1.

## Data Availability

Viral genomic sequences generated from this study were deposited in the NCBI Genbank under the accession numbers PP066138 to PP066243. The raw sequencing datasets are available in the NCBI Sequence Read Archive repository under the BioProject ID PRJNA1059154.

## Supplementary Materials

The following supporting information can be downloaded at: https://www.mdpi.com/article/10.3390/pathogens13020107/s1, Figure S1: Maximum likelihood phylogenetic tree of reoviruses; Figure S2: Maximum likelihood phylogenetic tree of orthoflaviviruses; Figure S3: Maximum likelihood phylogenetic tree of mesoniviruses; Figure S4: Maximum likelihood phylogenetic tree of iflaviviruses; Figure S5: Maximum likelihood phylogenetic tree of tombusviruses; Figure S6: Maximum likelihood phylogenetic tree of tymoviruses; Figure S7: Maximum likelihood phylogenetic tree of negev-like viruses; Figure S8: Maximum likelihood phylogenetic tree of *Orthomyxoviridae*; Figure S9: Maximum likelihood phylogenetic tree of *Phasmaviridae*; Figure S10: Maximum likelihood phylogenetic tree of *Rhabdoviridae*; Figure S11: Maximum likelihood phylogenetic tree of *Qinviridae* Table S1: Number of sequencing reads, contigs and viral reads for each mosquito pool.
