# Supplementary material for: Metatranscriptomic Sequencing of Medically Important Mosquitoes Reveals Extensive Diversity of RNA Viruses and Other Microbial Communities in Western Australia
Source: Pathogens. 2024 Jan 25;13(2):107. doi: 10.3390/pathogens13020107 (PMC10892203; doi:10.3390/pathogens13020107)
Supplement: Supplementary file 1 [file pathogens-13-00107-s001.zip › pathogens-2774923-supplementary.pdf]

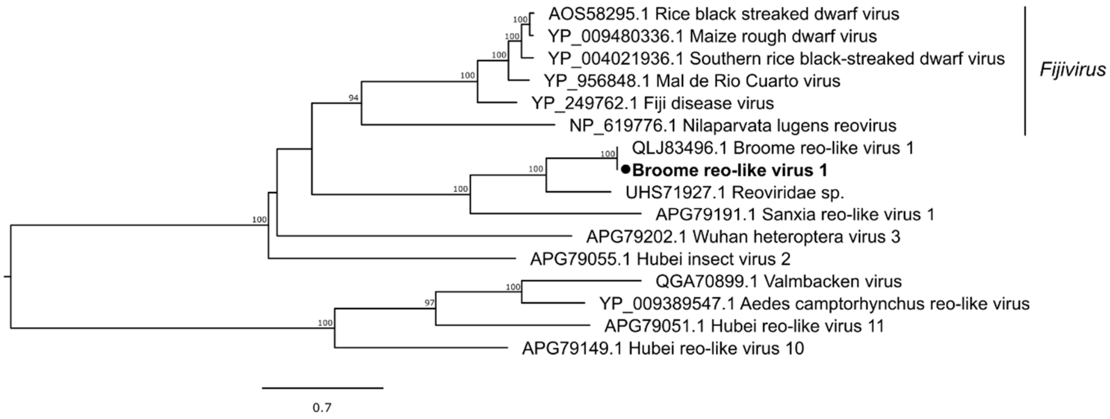

Figure S1. Midpoint rooted maximum likelihood phylogenetic tree of reoviruses showing the position of new variant identified (represented by a solid black circle). Bootstrap support values are displayed when greater than 70.

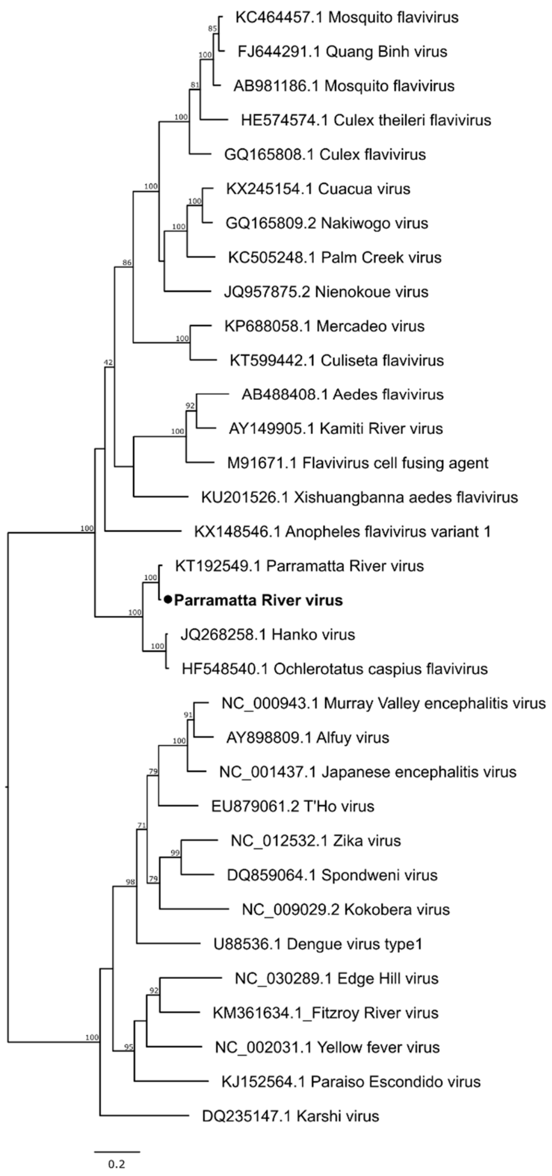

Figure S2. Midpoint rooted maximum likelihood phylogenetic tree of orthoflaviviruses showing the position of new variant identified (represented by a solid black circle). Bootstrap support values are displayed when greater than 70.

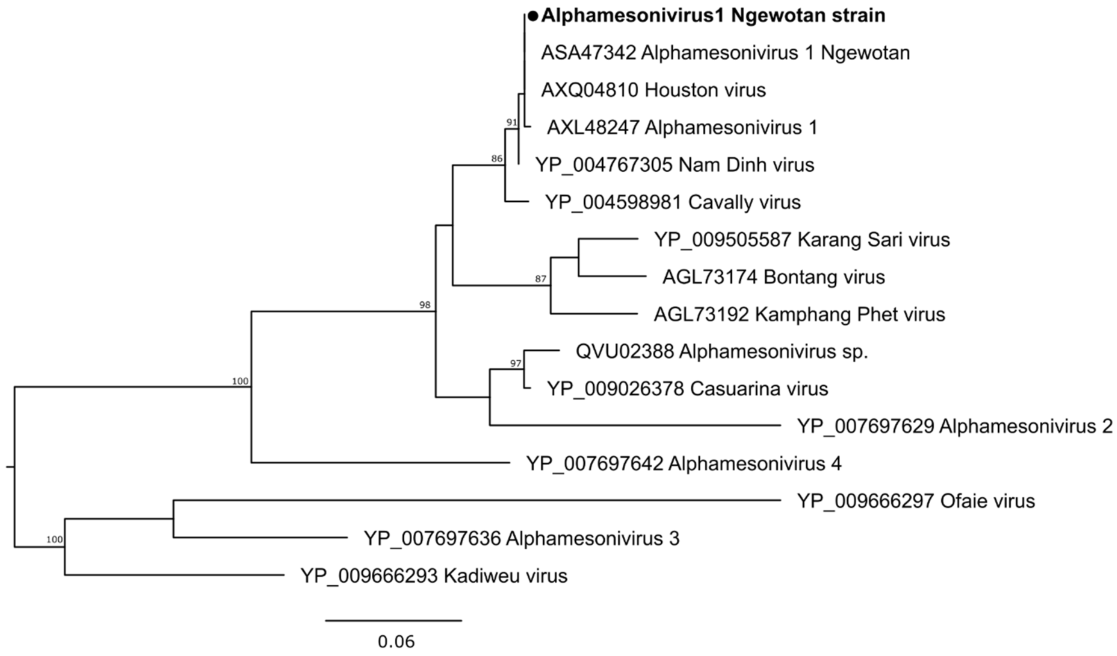

Figure S3. Midpoint rooted maximum likelihood phylogenetic tree of mesoniviruses showing the position of new variant identified (represented by a solid black circle). Bootstrap support values are displayed when greater than 70.

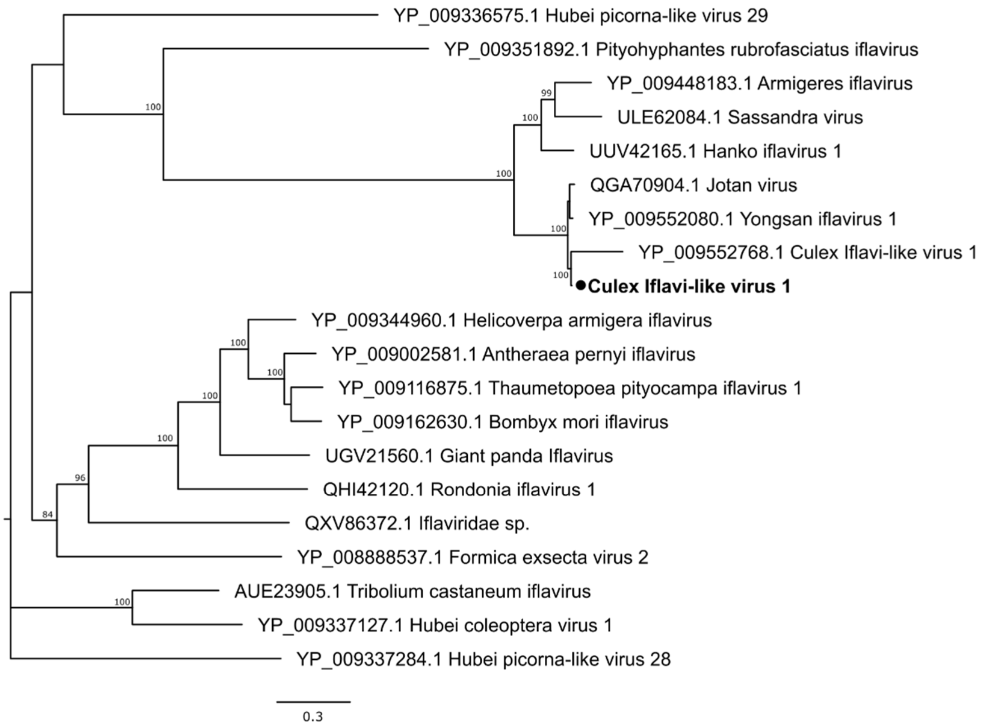

Figure S4. Midpoint rooted maximum likelihood phylogenetic tree of iflaviviruses showing the position of new variant identified (represented by a solid black circle). Bootstrap support values are displayed when greater than 70.

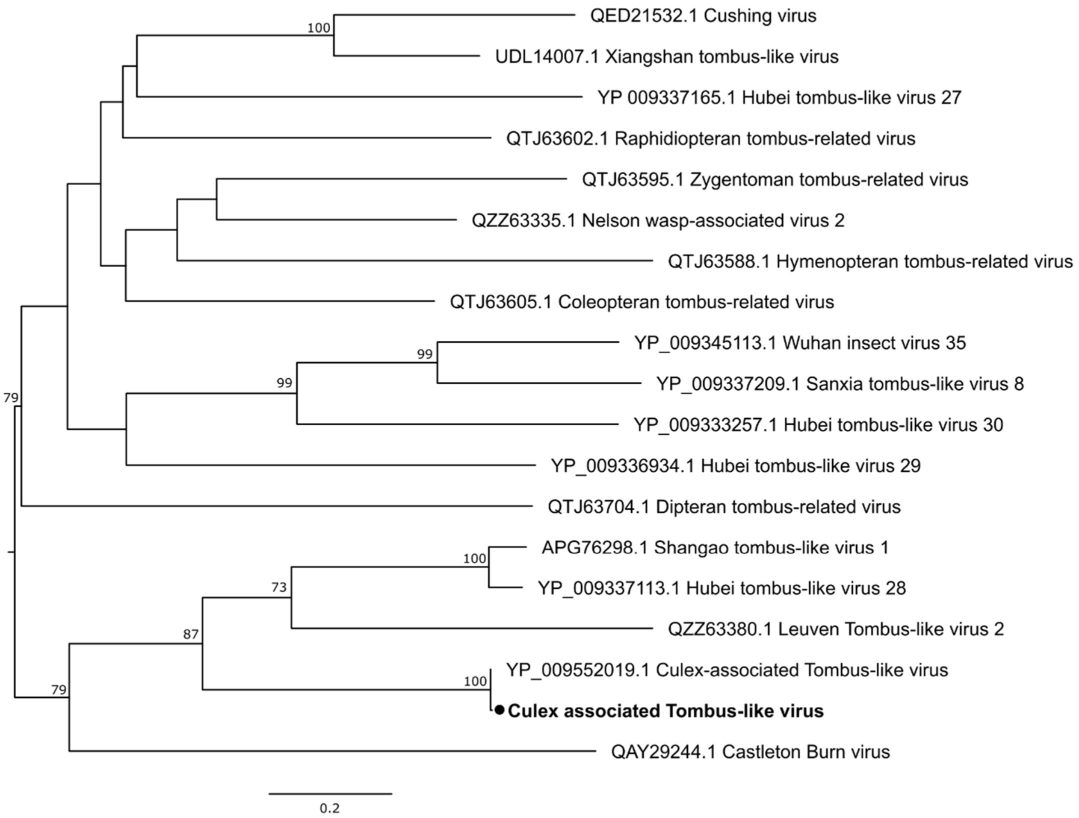

Figure S5. Midpoint rooted maximum likelihood phylogenetic tree of tombusviruses showing the position of new variant identified (represented by a solid black circle). Bootstrap support values are displayed when greater than 70.

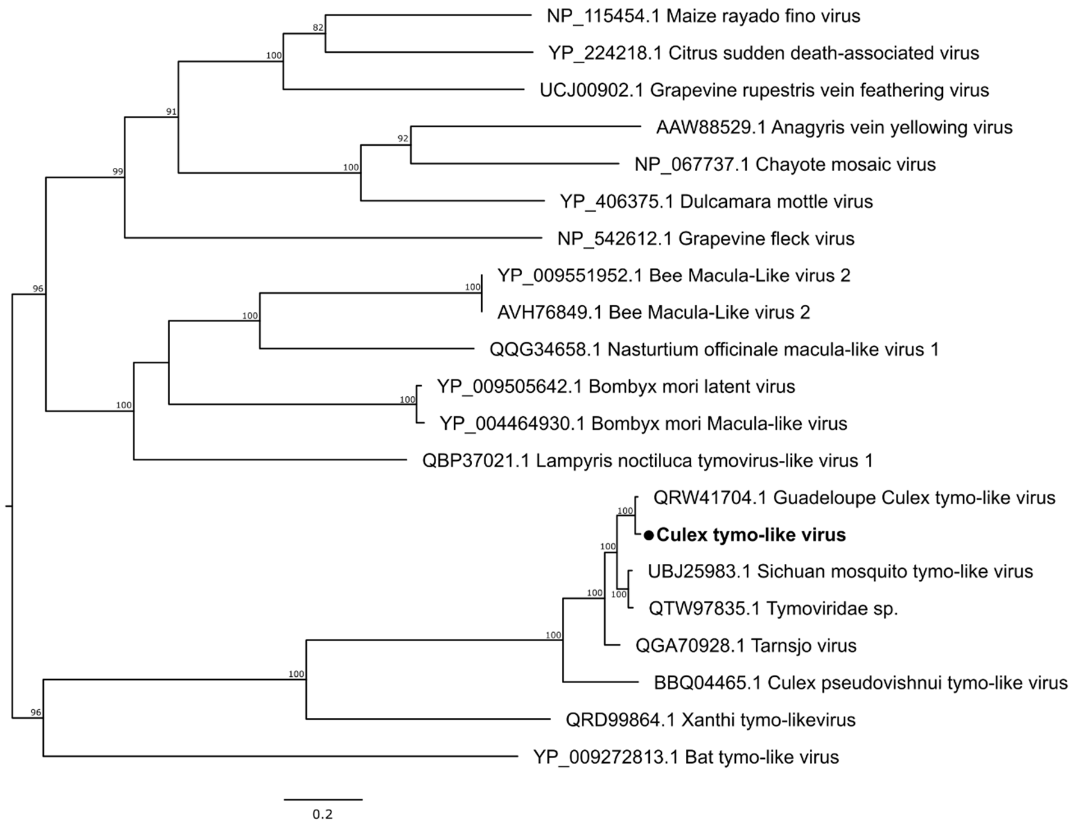

Figure S6. Midpoint rooted maximum likelihood phylogenetic tree of tymoviruses showing the position of new variant identified (represented by a solid black circle). Bootstrap support values are displayed when greater than 70.

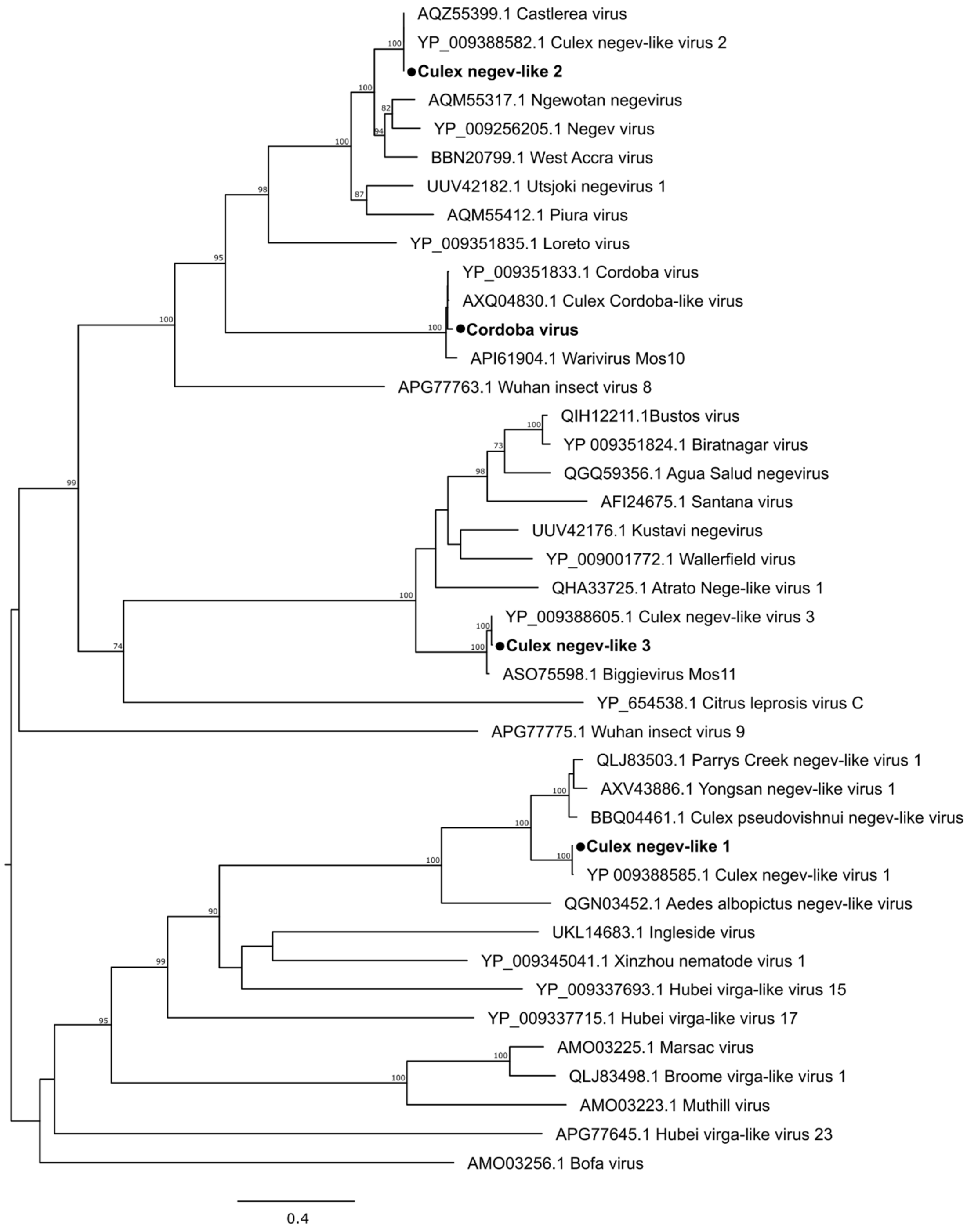

Figure S7. Midpoint rooted maximum likelihood phylogenetic tree of negev-like viruses showing the position of new variant identified (represented by a solid black circle). Bootstrap support values are displayed when greater than 70.

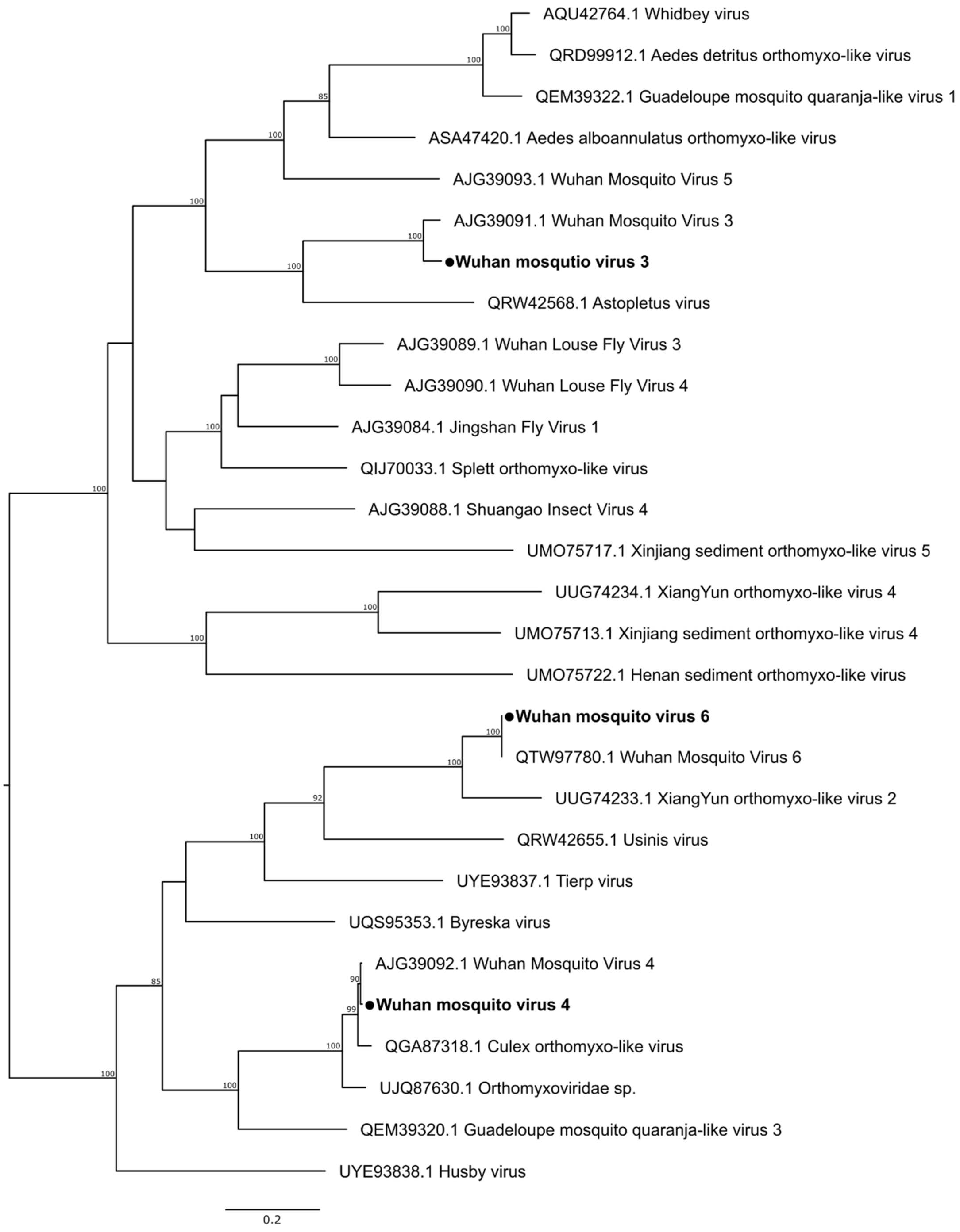

Figure S8. Midpoint rooted maximum likelihood phylogenetic tree of *Orthomyxoviridae* showing the position of new variant identified (represented by a solid black circle). Bootstrap support values are displayed when greater than 70.

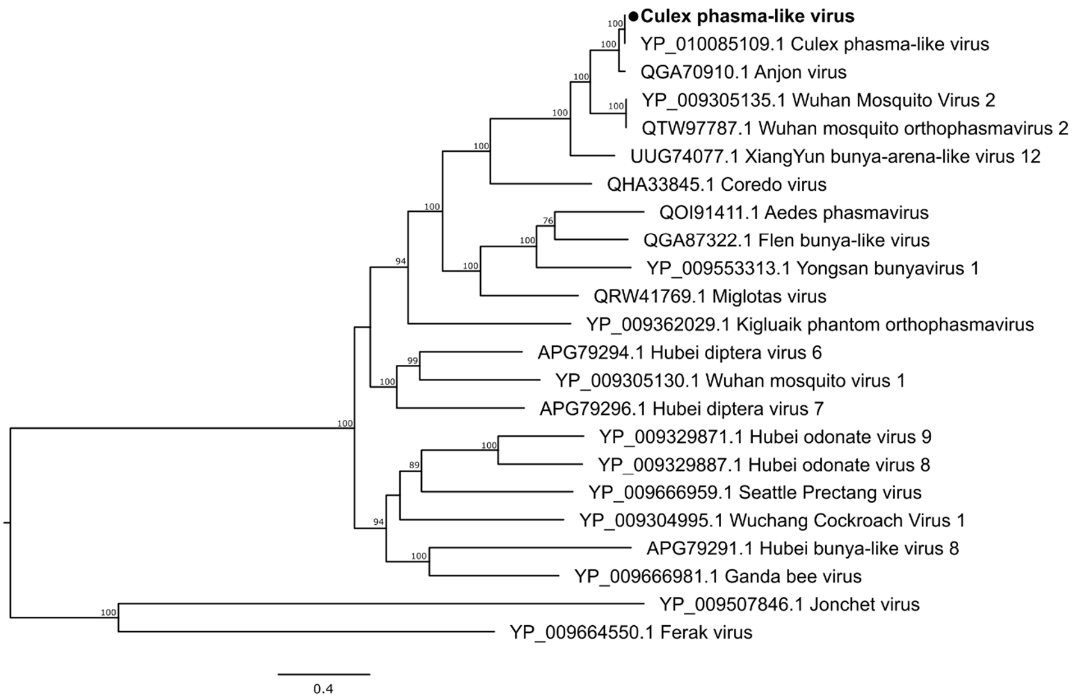

Figure S9. Midpoint rooted maximum likelihood phylogenetic tree of *Phasmaviridae* showing the position of new variant identified (represented by a solid black circle). Bootstrap support values are displayed when greater than 70.

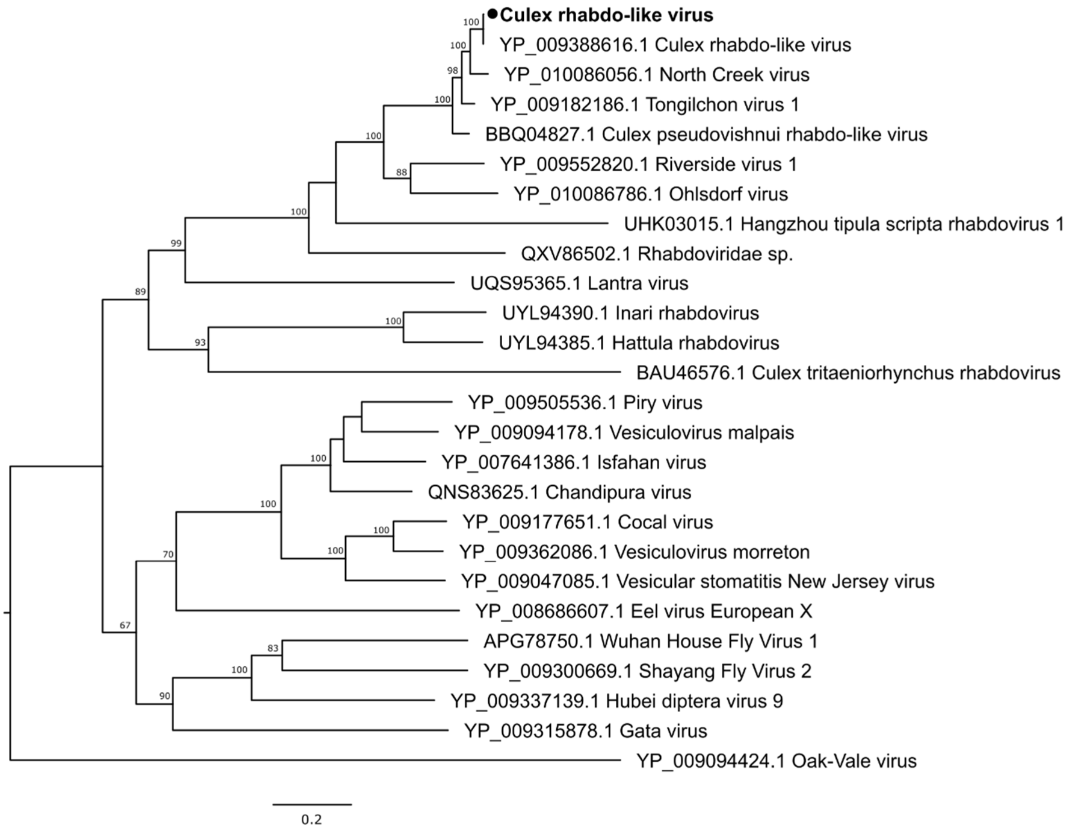

Figure S10. Midpoint rooted maximum likelihood phylogenetic tree of *Rhabdoviridae* showing the position of new variant identified (represented by a solid black circle). Bootstrap support values are displayed when greater than 70.

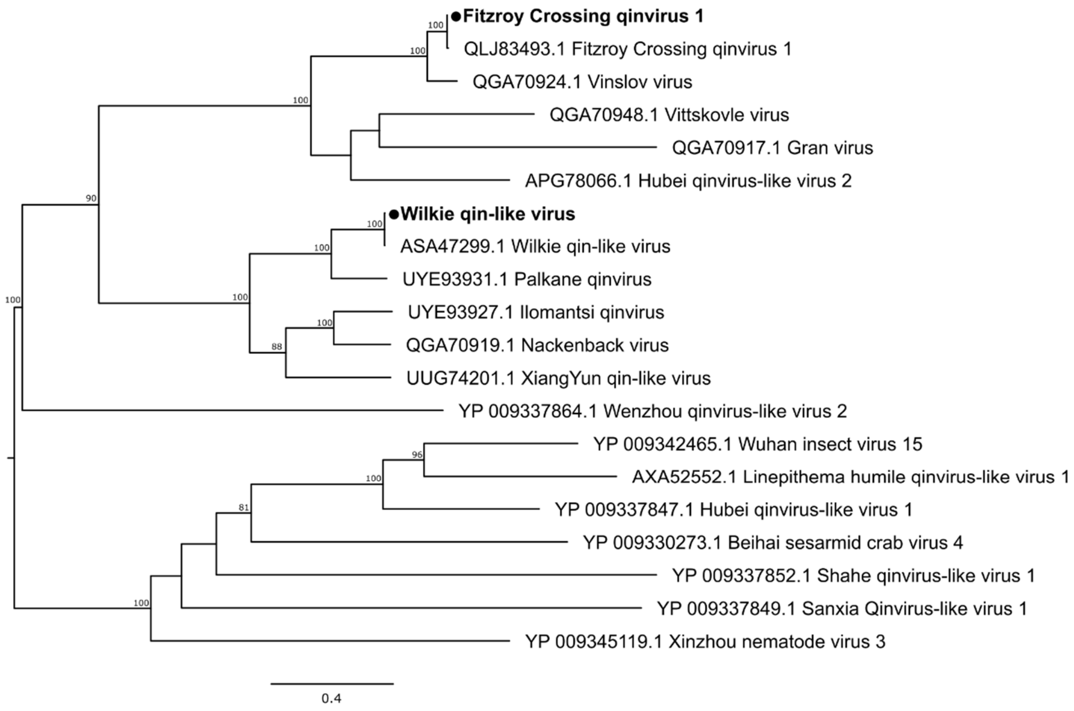

Figure S11. Midpoint rooted maximum likelihood phylogenetic tree of *Qinviridae* showing the position of new variant identified (represented by a solid black circle). Bootstrap support values are displayed when greater than 70.

Table S1. Number of sequencing reads, contigs and viral reads for each mosquito pool.

Mosquito Species	Total number of paired end reads	Total number of contigs	Total number of viral reads
Aedes vigilax Perth	84,573,893	41,679	1,171,387
Aedes vigilax Wyndham	85,644,338	48,447	63,542
Culex annulirostris Karrratha	85,648,932		36,016
Culex annulirostris Wyndham	183,824,887	135,094	280,507
Culex australicus	91,087,030	93,517	458,824
Culex globocoxitus	89,581,506	77,716	6,676,523
Culex pipiens biotype *molestus*	85,626,777	46,624	726,151
Culex quinquefasciatus	110,271,729	70,449	807,873
